# Immune-Modulating Effects of Conventional Therapies in Colorectal Cancer

**DOI:** 10.3390/cancers12082193

**Published:** 2020-08-06

**Authors:** Erta Kalanxhi, Sebastian Meltzer, Anne Hansen Ree

**Affiliations:** 1 Department of Oncology, Akershus University Hospital, 1478 Lørenskog, Norway; ekalanxhi@outlook.com (E.K.); sebastian.meltzer@medisin.uio.no (S.M.); 2 Institute of Clinical Medicine, University of Oslo, 0318 Oslo, Norway

**Keywords:** colorectal cancer, high-risk, metastasis, oxaliplatin, radiation, immune checkpoint blockade

## Abstract

Biological heterogeneity and low inherent immunogenicity are two features that greatly impact therapeutic management and outcome in colorectal cancer. Despite high local control rates, systemic tumor dissemination remains the main cause of treatment failure and stresses the need for new developments in combined-modality approaches. While the role of adaptive immune responses in a small subgroup of colorectal tumors with inherent immunogenicity is indisputable, the challenge remains in identifying the optimal synergy between conventional treatment modalities and immune therapy for the majority of the less immunogenic cases. In this context, cytotoxic agents such as radiation and certain chemotherapeutics can be utilized to enhance the immunogenicity of an otherwise immunologically silent disease and enable responsiveness to immune therapy. In this review, we explore the immunological characteristics of colorectal cancer, the effects that standard-of-care treatments have on the immune system, and the opportunities arising from combining immune checkpoint-blocking therapy with immune-modulating conventional treatments.

## 1. Introduction

Colorectal cancer (CRC) is the third-most commonly diagnosed cancer worldwide with 1.4 million new cases annually [1], contributing to the fourth-most common cancer-related deaths [2]. Age is considered to be the strongest risk factor with the majority of cases being diagnosed in those aged over 50, and with a significant rise in incidence from the age of 60. CRC is more prevalent in developed, Western countries where the average lifetime risk of 3–5% rises significantly in individuals with a family history of CRC [2].

Even though the majority of CRC cases are of a sporadic nature, a subgroup of patients presents with hereditary forms of CRC, such as the common manifestation of Lynch syndrome or the rare familial adenomatous polyposis. In individuals with the former, inherited mutations or epigenetic inactivations of DNA mismatch-repair (MMR) genes result in a deficient MMR (dMMR) function. For those developing CRC, MMR deficiency usually causes a high tumor mutational burden or microsatellite-instable (MSI) status [3]. Apart from Lynch syndrome, sporadic cases of early-stage dMMR tumors contribute to a total population of approximately 15% of CRC cases and are associated with better long-term outcomes than those with proficient MMR (pMMR) function [4].

A quarter of all CRC patients present with metastatic disease at the time of diagnosis, whereas approximately half of the total patient population will eventually have developed metastases. The most frequently affected distant organ is the liver, followed by the lungs and peritoneum [5]. The prevailing theory of metastatic progression in cancer is the classic seed-and-soil concept, and CRC liver metastasis (CLM) is perfectly explained by this theory as the mesenteric venous drainage filters directly to the liver. Regarding primary tumors of the lower rectum, the inferior rectal vein communicates to the inferior vena cava, making these tumors more prone to metastasize to the lungs [6,7].

Generally, no curative treatment exists for multi-organ metastasis. In the past two decades, a number of new systemic therapies of cytotoxic and biologically targeted agents have been taken into routine use for advanced CRC, extending patients’ lives and most importantly, alleviating their symptoms. Nevertheless, unresectable metastases, particularly in abdominal cavity organs, remain the cause of severe morbidity and poor survival [8]. New insights into the underlying biology of systemic tumor dissemination may guide the next milestone in CRC management, the control of metastatic progression.

Within this frame of reference, the potential of using the immune system to fight progressing cancer has opened paradigm-changing therapeutic avenues. Tumor-defeating immunity involves both tumor-antigen recognition and the action of cytotoxic T-lymphocytes. However, as the tumor is also “self”, protective mechanisms against auto-immunity impede immune surveillance. This counterbalance between the cancer and the immune system creates a state of equilibrium, or immune tolerance, which can be edited therapeutically. Therefore, immune therapies are aimed at overcoming tumor-induced immune-suppressive mechanisms and invoking antitumor immune responses. So far, this concept has proven successful in the treatment of a limited number of immunogenic tumors, but for less immunogenic cancers such as the majority of CRC cases, additional stimulation is required to breach the immune tolerance and for patients to achieve beneficial and durable treatment responses.

## 2. The Tumor Microenvironment (TME)

### 2.1. The Hypoxic TME and Its Immune Attributes

Hypoxia (low tumor oxygenation), a major TME (tumor microenvironment) hallmark, continuously selects for tumor cell clones with aggressive phenotypes and stimulates epithelial-to-mesenchymal transition, a process during which tumor cells lose the ability to adhere to each other in favor of increased migration within the extracellular matrix [9]. Hypoxic conditions promote resistance to cytotoxic therapies and contribute to metastatic progression [10,11,12]. Furthermore, inflammation in the hypoxic TME provokes immune tolerance via tumor- and T-cell expression of immune checkpoint proteins [13,14]. For example, increased expression of programmed death receptor 1 (PD-1) ligand, PD-L1, in tumor cells renders them more effective in binding to PD-1 on cytotoxic T-cells, thereby silencing T-cell activity. On the other hand, immune checkpoint-blocking antibodies such as anti-PD-1 and anti-PD-L1 agents, approved for treatment of immunogenic tumors such as melanoma, classic Hodgkin lymphoma, and others, act by unleashing the cytotoxic T-cell activity because they deblock the inhibitory binding of the PD-L1 on tumor cells to the PD-1 immune checkpoint T-cell receptors. Thus, this phenomenon is commonly referred to as immune checkpoint blockade (ICB).

Studies have demonstrated major responses and long-term survival to ICB in early-stage, locally advanced, and metastatic dMMR CRC, characterized by high tumor mutational burden and positive MSI status and therefore high densities of tumor-associated antigens [15,16,17,18,19]. Additionally, strong ICB responsiveness has been observed in advanced CRC with a rare gene locus copy-number gain that causes enhanced PD-L1-mediated immune checkpoint activity [20]. However, unlike the case for melanoma and non-small-cell lung cancer, tumor-cell expression of PD-L1 has not shown utility as a predictive biomarker for ICB response in CRC [21]. Because the efficient activation of tumor-defeating T-cells requires their neo-antigen priming by antigen-presenting dendritic cells, the dominant population of CRC patients with pMMR disease and low tumor mutational burden is inherently non-immunogenic [22,23]. In theory, either the eradication of TME hypoxia or the enhanced release of tumor-associated antigens may breach the immune tolerance, a notion supported by recent findings [24] where CRC patients with poor prognosis in any disease stage had tumors that were concurrently hypoxic and sparse in stromal T-cells and displayed an epithelial-to-mesenchymal transition molecular signature [25].

### 2.2. Molecular CRC Subtypes

The heterogeneous nature of CRC at the molecular and clinical level has warranted a more comprehensive method of classification that combines molecular signatures of tumor cells with TME features (immune cells and other stromal components) [22]. The consensus molecular subtype (CMS) classification, which is the one most commonly used, is based upon data from six different classification systems and categorizes CRC primary tumors into four different subtypes (CMS1-4) with varying degrees of mutations, immune-cell infiltration, metabolic activations, and other TME changes [26]. For example, CMS1, also referred to as the Immune Subtype, applies to 14% of cases and includes hypermutated tumors with a high degree of immune-cell infiltration and strong immune activation. CMS4, also referred to as the Mesenchymal Subtype, is characterized by upregulation of genes involved in the epithelial-to-mesenchymal transition, angiogenesis, and matrix remodeling. The CMS classification has been validated in preclinical models and holds promise with respect to clinical utility [27,28].

### 2.3. The CRC Immunoscore

It has been over a decade since Galon and colleagues elegantly demonstrated that the tumor’s immune–cell contexture, as assessed in large cohorts of CRC patients with early-stage disease, was better at predicting patient survival than well-established histopathological methods [29]. This contexture, which describes the type, density, and location of adaptive immune cells in the TME, varies greatly between individual patients [30]. Specifically investigated in localized CRC, both tumor infiltration of cytotoxic T-cells and molecular T-cell signatures were correlated with the absence of histological signs of invasiveness and good clinical outcome [29,31]. This work was further expanded to show that the Immunoscore, a quantification of cytotoxic T-cells in the tumor core and invasive margin, exceeded the capability of positive MSI status and was independent at predicting disease-free survival in patients undergoing primary tumor resection [30,32,33]. Finally, an international consortium consisting of 14 centers in 13 countries assessed samples from more than 2600 patients with localized colon cancer by standardized protocols for immunohistochemistry and digital image analysis, and validated that the Immunoscore was independent of factors such as age, sex, TN-stage, and MSI status at predicting disease-free survival [34]. The prognostic power of the Immunoscore was recently confirmed within a prospective adjuvant chemotherapy study [35]. Furthermore, it has been approved as an in vitro diagnostic device in clinical practice but at present without reimbursement by the public health services [36].

However, it has been argued that the Immunoscore fails to include other immune-cell populations hosted by the TME such as macrophages, dendritic cells, and natural killer cells, all of which may also be important for predicting prognosis [37]. These cell types have been studied extensively in predominantly early-stage CRC, where high infiltration within the tumor, and peripherally in the case of dendritic cells, is associated with favorable prognosis [38,39,40,41]. Paradoxically, this is also true for regulatory T-cells, which are central in the maintenance of immune tolerance [39,41]; however, this may be due to an alternative T-regulatory population that participates in immune surveillance [42]. To date, the Immunoscore and CMS classification have not been integrated nor evaluated head-to-head with regard to clinical utility by the respective developers. In T1 CRC (the most superficially invasive tumors with excellent prognosis and consequently few CMS4 cases), no significant association was found between the Immunoscore and CMS subtypes [43].

## 3. Immune Modulation by Cytotoxic Therapies

### 3.1. Fluoropyrimidines

This class of agents constitutes the mainstay of most CRC treatment regimens. Their mechanism of action relies on disruption of DNA synthesis by the incorporation into DNA and RNA structures and by inhibition of the DNA synthesis enzyme thymidylate synthase [44]. The most commonly used fluoropyrimidine over the years is fluorouracil, which is usually given with folinic acid to increase its affinity for thymidylate synthase. With respect to its immune-related effects, fluorouracil is shown to have an ambivalent role through its selective targeting of myeloid-derived suppressor cells (MDSC), an innate immune-cell population. By targeting MDSC, fluorouracil lifts some of the inhibition that MDSC impose on T-cell activation, but at the same time, the activation of the inflammasome complex in the dying MDSC leads to the release of pro-inflammatory cytokines that ultimately promote angiogenesis and tumor growth [45,46]. Additionally, an in vitro study showed that fluorouracil increased PD-L1 expression in CRC cell lines [47].

### 3.2. Oxaliplatin

This agent is a third-generation platinum analog. The mechanism behind its cytotoxicity relies on the adduct formation with DNA, resulting in the blocking of DNA replication and transcription. Reduction of thymidylate synthase synthesis is considered as a secondary effect of oxaliplatin that contributes to the synergy observed when oxaliplatin is combined with fluorouracil [48].

**Figure 1:**
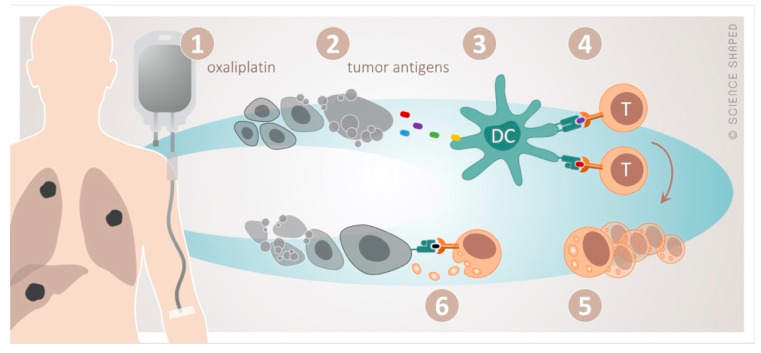
The concept of immunogenic cell death by oxaliplatin. Cytotoxic damage by oxaliplatin (1) causes release of tumor antigens from the dying tumor (2). These are taken up (3) by dendritic cells (DC) and presented to cytotoxic T-cells (4), resulting in their activation and clonal proliferation (5). This will in principle enable specific T-cell-targeting of any tumor manifestation systemically (6).

Oxaliplatin perfectly illustrates the interplay between cytotoxic and immune-modulating effects of chemotherapy, the latter in terms of immunogenic cell death (ICD) [49,50] (Figure 1). In the dying tumor cell, ICD is preceded by the translocation of calreticulin, an endoplasmic reticulum stress response protein, to the plasma membrane and the extracellular release of ATP and the high-mobility group box-1 (HMGB1) protein. Calreticulin serves as an “eat me” signal, ATP recruits dendritic cells to the tumor sites to cause their maturation to antigen-presenting cells, and HMGB1 facilitates the Toll-like receptor 4 dependent cross-presentation of the released tumor antigens to cytotoxic T-cells.

The ability of oxaliplatin to initiate ICD may play a critical role with respect to its rational combination with ICB therapy. Preclinical studies demonstrated that oxaliplatin provoked TME infiltration of cytotoxic T-cells and sensitization of CRC and other adenocarcinomas to ICB therapy [51,52,53]. Another study using a CLM model showed that oxaliplatin (in cooperation with interleukin-12) reduced the TME content of immune-suppressive T-regulatory cells and MDSC independently of HMGB1 and calreticulin release [54].

Another platinum analog, cisplatin, was unable to sensitize CRC models to ICB therapy [51]. Interestingly, both oxaliplatin and cisplatin induced the release of HMGB1, but it was only oxaliplatin that caused calreticulin translocation and effectively contributed towards tumor immunogenicity [55]. Moreover, HMGB1 binding to Toll-like receptor 4 seemed to be critical for the oxaliplatin efficacy, since patients carrying a loss-of-function TLR4 allele had impaired progression-free survival (PFS) when given oxaliplatin for metastatic disease [55].

### 3.3. Radiation

Approximately 50% of cancer patients receive radiotherapy during the disease course [56]. Ionizing radiation is cytotoxic in the sense that DNA damage in the targeted cells causes senescence or cell death by means of apoptosis, necrosis, autophagy, or mitotic catastrophe [57]. In addition to the release of tumor-associated antigens, radiation also provokes the release of calreticulin and ATP, leading to the recruitment and activation of dendritic cells. As such, radiation can be viewed as an in situ vaccine with the potential to induce tumor regression systemically [58]. Systemic effects of radiation, away from the radiotherapy target volume, constitute the phenomenon known as abscopal effect. Even though abscopal events are rare, they have been observed both in experimental and clinical settings [58,59]. The clinical manifestation of the abscopal effect occurs when radiotherapy has been administered together with factors that enhance the antitumor immune response, such as the dendritic-cell stimulator granulocyte-macrophage colony-stimulating factor or ICB therapies [59,60,61,62,63,64,65]. The majority of studies combining radiation with ICB therapy have utilized single-site irradiation, a setting that has been recently proposed to be partially responsible for low response rates. The heterogeneity of tumor-associated antigens in the metastatic lesions and the varying degrees of immunogenicity generated by different organs may warrant irradiation at different sites in order to achieve an adequate immune response [66,67].

However, not all radiation effects are in favor of the antitumor response, and radiation has been shown to also enhance TME immune-suppressive features in experimental models. For example, dendritic cells co-cultured with irradiated murine mammary carcinoma cells had significantly reduced surface expression of both antigen-presenting and co-stimulatory markers [68]. Transforming growth factor-β1, one of the major immune-suppressive factors induced by radiation, contributes to inhibition of T-cell cytotoxicity [69], immune evasion [70], expansion of T-regulatory cells [71], inhibition of dendritic cell activation, as well as to the induction of pro-tumorigenic phenotypes in both tumor-associated neutrophil and macrophage populations [72,73]. This complex role of radiation in immune responses emphasizes the necessity for combination treatments aimed at enhancing the synergy among the various modalities. Preclinically, radiation has been shown to enhance pro-immunogenic responses for both ICD-inducing chemotherapeutic agents and immune checkpoint inhibitors [74,75]. Importantly, radiation dose and fractionation are determinative for suppressive or activating effects; therefore, dose and sequence of delivery, especially in conjunction with other modalities, should be considered [76]. For example, in a mammary carcinoma mouse model, irradiation with high doses induced a DNA exonuclease, which through degradation of immunogenic DNA fragments accumulating in the cytosol in irradiated cells caused a decline in the recruitment of dendritic cells crucial for T-cell-mediated systemic tumor rejection. However, lower dose fractions did not result in the exonuclease induction [77].

## 4. Immune-Modulating Opportunities in the Standard-of-Care

### 4.1. Cytotoxicity and Tumor-Defeating Immunity

In principle, three scenarios can be set for “on-target” cytotoxicity combined with “off-target” tumor-targeting immune responses in the standard-of-care CRC management. They are: neoadjuvant treatment of locally advanced disease in order to impede metastatic progression, neoadjuvant treatment of oligometastatic disease in order to achieve systemic tumor clearance, and early-line therapy in unresectable metastatic disease for durable systemic control.

### 4.2. Neoadjuvant Treatment of Locally Advanced Disease

In locally advanced rectal cancer (LARC), neoadjuvant chemoradiotherapy (CRT) with a fluoropyrimidine in a non-cytotoxic radiosensitizing dose and resection of the residual tumor result in low local recurrence rates [78], but metastatic progression remains a dominant cause of failure [79,80]. While there is no consensus whether postoperative systemic therapy may reduce metastatic risk [79,81,82,83], various regimens involving neoadjuvant chemotherapy (NACT) prior to or immediately following radiation have been investigated. Specifically, oxaliplatin-/fluoropyrimidine-based NACT has been administered before CRT (in trials not randomized for the induction therapy) or following short-course radiation prior to surgery (the experimental arm of the RAPIDO trial) with the aim of delivering adequate systemic therapy for metastasis prevention without compromising local disease control [84,85,86,87,88,89,90,91,92]. Because radiation has the ability to deliver cytotoxic effects in a focused tumor volume, it has been argued that improved systemic outcome may be achieved by intensifying the local effects that enhance elimination of clonogenic cells [93]. In that regard, only two of seven randomized studies that have evaluated the potentially radiosensitizing effect of concomitant oxaliplatin in fluoropyrimidine-based CRT met the primary efficacy endpoint [80,94,95,96,97,98,99]. The same two studies showed high patient compliance to the chosen dose scheduling of oxaliplatin [80,95]. It is tempting to speculate that oxaliplatin-based neoadjuvant therapy, when administered at an intensity that does not compromise patient compliance to the multimodal treatment protocol, may promote an abscopal immune response [100].

### 4.3. Neoadjuvant Treatment of Oligometastatic Disease

In liver-confined metastatic CRC with a therapeutic intent for surgical resection or radiofrequency ablation, oxaliplatin-based NACT can be used for patient selection with respect to tumor aggressiveness, disease down-sizing, or conversion of initially unresectable disease [101], three sides to the same story. Although the EPOC trial showed no overall survival (OS) benefit for patients randomized to perioperative oxaliplatin-based chemotherapy compared to those receiving only surgery, perhaps explained by the study power to investigate PFS and the inclusion of more than 50% of patients with a single metastatic lesion, the combined-modality study arm has become the standard-of-care [101,102,103]. A correlative examination of the surgical specimens revealed that the neoadjuvant therapy caused tumor infiltration of CD3-positive lymphocytes and that a high count of the CD3-positive cells at the invasive margin between tumor and the liver tissue was beneficial for PFS [104]. In terms of response rates of initially unresectable CLM, the best outcomes in randomized trials were observed for regimens containing irinotecan or cetuximab in addition to oxaliplatin-based NACT [105,106], with improved long-term outcomes for both resected and non-resected patients reported for the former regimen [107,108]. Meta-analyses have been discordant regarding survival benefit of perioperative chemotherapy in patients with initially resectable CLM [109,110].

Hepatic artery infusion (HAI) chemotherapy is attractive as it limits systemic toxicity and allows combination with systemic therapy. A number of early-phase trials have investigated the concept, using oxaliplatin as the HAI compound for unresectable CLM with treatment toxicity and response as endpoints, but only one study has been carried on with the aim of transforming to resectable disease by using oxaliplatin, irinotecan, and fluorouracil [111]. The investigators have also examined systemic exposure of the HAI chemotherapeutics in terms of pharmacokinetics analyses [112] and pharmacogenetics determinants of outcome [113].

It is clear that immune responses arise from the ”off-target” effects of these various treatment approaches. However, their involvement towards the eradication of occult tumor at other sites (abscopal effect) has been essentially unexamined. Thus, we studied antitumor immunity invoked by oxaliplatin-HAI and the long-term outcome of primarily unresectable CLM in patients who had their first recurrence of oxaliplatin-naïve isolated CLM that was considered technically unresectable [114]. Those who presented a rapid and substantial rise in a circulating ICD factor over the initial treatment and at its completion could proceed to CLM ablation (hepatic resection or radiofrequency ablation) or had complete response, were alive at final follow-up 8–12 years later, which is a remarkable outcome. In contrast, those who remained with technically unresectable CLM or had the disease converted to resectability but presented a slow and gradual accretion of the ICD factor, later died of the metastatic disease. Consequently, complete and durable tumor eradication appeared to be contingent on CLM conversion to resectable disease (for macroscopic tumor clearance) along with a strong antitumor immune response (for elimination of disseminated microscopic tumor cells that might cause systemic failure).

### 4.4. Early-Line Therapy in Unresectable Metastatic Disease

Patients with disseminated CRC make up an extremely heterogeneous group, with survival rates depending on the metastatic sites, tumor load, and severity of systemic inflammation; however, median OS has improved to approximately 30 months in clinical trials [115]. The powerful ICB effects with respect to tumor regression rates and disease control durability in patients with dMMR/MSI-high CRC have not been seen in pMMR cases. A variety of studies that investigate strategies to enhance cytotoxic T-cell activation and tumor infiltration in combination with ICB therapy are ongoing [116], and the first reports on outcomes can soon be expected [117,118]. Examples of how to combine ICB with immune-modulating conventional therapies will be discussed below. If such attempts succeed to become early-line treatments of metastatic disease, advanced CRC may be converted into a lifelong controllable illness for the dominant pMMR patient population.

## 5. High-Risk Rectal Cancer—A Case Study

### 5.1. Blood-Based Indicators of Treatment Outcome and Tolerance

As conveyed in the above sections, the effects that cytotoxic therapies have on the immune system are complex and often hanging on a balance between activating and suppressive responses. Despite the accumulating preclinical evidence displaying synergies between cytotoxic and immune therapies, the heterogeneous nature of cancer as it progresses and the individual differences of the hosts’ immune systems present practical barriers that need to be overcome for this partnership to be clinically successful. A better biological insight into the individual patient’s systemic responses to conventional treatment is an important step towards a rational integration of immune therapy in this context. Conceptually, detection of circulating proteins that may reflect the changing TME and its systemic manifestations, as well as the constitutional and acquired physiology of the patient, is a promising path to treatment-related biomarker discovery. We employed an antibody array technology to monitor serial serum samples collected throughout an intensified neoadjuvant treatment course in LARC study patients, in order to dissect the contribution of the individual modalities to treatment outcome and tolerance [119]. The commercial antibody array detected approximately 500 proteins that included immune factors, epithelial and vascular growth factors, and proteinases. Alterations in serum levels of these proteins were anticipated to reflect treatment-induced effects on the tumor mass and other tissues. The advantage of simultaneously analyzing a myriad of circulating proteins on an array lies in the ability to achieve a more complete picture of the patient’s systemic responses to the treatment.

### 5.2. The Clinical and Translational Study Design

The particular study (NCT00278694) prospectively enrolled 97 patients with T2-4N0-2M0 rectal cancer between 2005 and 2010, who were given oxaliplatin-/fluorouracil-containing induction NACT and sequential CRT with concomitant oxaliplatin weekly and capecitabine daily on the days of radiotherapy before pelvic surgery [120,121]. The study population consisted of cases that were considered high-risk: the T2 cases presented a primary tumor threatening the anal levator muscles; the T3 cases had mesorectal fascia margin of 2 mm or less; the T4 cases had organ-infiltrating tumor; the majority of patients had involved lymph nodes in the pelvic cavity. In LARC, it is increasingly appreciated that the sequence and combination of the various treatment modalities should be optimized in accordance with the patient’s risk stratification; hence, the aim of this study on high-risk patients was to deliver systemic therapy in the neoadjuvant setting and to intensify local radiation effects. However, since protraction of the total treatment time may theoretically permit tumor cell repopulation and selective pressure towards the survival of therapy-resistant cell clones, only four weeks of the induction NACT (two cycles of the Nordic FLOX regimen [122]) were given. This induction phase was highly tolerable [120] and alone led to substantial tumor volume reduction [123]. Moreover, an adapted oxaliplatin dose intensity during CRT maintained patient compliance to the radiotherapy protocol [100]. In this study population with a high proportion of T4 disease and lymph node involvement, 5-year PFS (almost all PFS events were metastatic progression) and OS were remarkably good [120], an observation that prompted us to explore the possible mechanisms involved.

The study biobank consists of serum and plasma samples collected at the time of diagnosis, immediately following NACT and then CRT completion, and at the time of treatment evaluation four weeks after the neoadjuvant treatment. Following the initial screening with the antibody array [119], we systematically investigated circulating factors likely reflecting the therapeutic eradication of TME hypoxia and local invasiveness as well as immune-related responses: carbonic anhydrase 9 (CA9), matrix metallopeptidase 9 (MMP9), osteoprotegerin (OPG; formally denoted TNFRSF11B), and fms-related tyrosine kinase 3 ligand (FLT3LG) [100,119,124,125].

#### 5.2.1. CA9

The almost exclusively tumor-specific CA9, whose gene is induced by the hypoxia-inducible factor-1α, is a transmembrane enzyme aimed at controlling intracellular pH changes that result from the accumulation of acidic metabolites under hypoxic conditions. Specifically, CA9 catalyzes the reversible hydration of carbon dioxide to bicarbonate and protons, which restores physiological intracellular pH [126]. However, as this enzymatic reaction normalizes intracellular pH, inefficient metabolic waste clearing from the abnormal TME vasculature leads to TME acidification. This induces metallopeptidase activation with remodeling of the extracellular matrix and enhanced tumor-cell invasiveness, which may be one of the links between high tumor CA9 expression and poor prognosis [127,128]. Active metallopeptidases further cause proteolytic CA9 cleavage and consequently its ectodomain shedding [129]. We therefore measured circulating CA9 in the study patients to investigate any possible association of CA9 release with therapeutic outcome [124].

We found that patients who from their individual baseline level showed a strong increase in circulating CA9 after completion of the short-course induction NACT, had significantly better survival without metastatic progression than patients with low post-NACT versus baseline alteration. Strikingly, this strong CA9 increase was significantly correlated with tumor down-staging and node sterilization, but not with the degree of surviving tumor cells relative to fibrosis (i.e., tumor regression grade) of the surgical specimen [124]. The histological observations raised the speculation that a response from CA9-expressing TME stromal cells to the induction NACT [130], in addition to the response from the actual tumor cells, might have been conditional for the favorable survival outcome. This entire set of observations further indicated that the oxaliplatin-containing chemotherapy might have specifically targeted hypoxic, CA9-expressing tumor components.

#### 5.2.2. MMP9

This metallopeptidase belongs to a family of proteases with critical roles in extracellular matrix degradation and remodeling as well as angiogenesis [131]. Lipocalin-2, which is predominantly produced in neutrophils, forms a covalent complex with MMP9 and protects it from auto-degradation [132]. MMP9 production occurs in both TME stromal and inflammatory cells such as macrophages and neutrophils [133,134], the latter storing MMP9 in secretory granules for rapid release [135]. Circulating MMP9 is elevated in CRC patients, and in resected primary CRC, tumor MMP9 expression is an independent predictor of disease-free survival [136,137,138,139].

We found that circulating MMP9, together with lipocalin-2, had significantly declined from the baseline values at completion of the induction NACT and gradually reverted over the remaining neoadjuvant course. Moreover, the greater the fall in post-NACT and post-CRT MMP9 levels, the more favorable survival without metastatic progression [119]. These observations made it tempting to speculate that the induction NACT, aided by the sequential CRT, might have specifically eradicated TME components with MMP9-regulated inflammatory and invasive properties [140] in good-prognosis patients. Additionally, the serum MMP9 decline might have reflected a myelosuppressive effect of NACT and CRT (i.e., a systemic adverse effect on neutrophils) as we could not exclude the possibility that MMP9 (and lipocalin-2) alterations might mirror deleterious normal tissue effects. With the particular study design, the pelvic CRT caused enteritis [96], but we did not find any correlation between the actual factors and diarrhea scores, the clinical correlate of adverse intestinal effects [119].

#### 5.2.3. OPG

This soluble tumor necrosis factor decoy receptor is a glycoprotein that binds to the ligand of the receptor activator of nuclear factor-kappaB (RANK), RANKL [141]. The OPG/RANKL/RANK system is involved in a wide variety of biological processes and is essential for bone-resorbing osteoclast activity of the bone remodeling process [142,143]. In particular, RANKL-induced signaling is implicated in the antigen-specific interaction between dendritic cells and T-cells [143].

We found that circulating OPG appeared to show contradictory biological behavior. On the one hand, high baseline levels were associated with metastatic progression after the pelvic surgery [125]. It might be that high de novo circulating OPG reflected a rescue mechanism to high osteoclast activity that is commonly associated with disease of grave severity. On the other hand, patients who responded to the induction NACT by elevating the circulating OPG, had considerably better metastasis-free survival than those who did not achieve a rise, even though patients with and without increase were equally distributed with regard to tumor down-staging and node sterilization as well as tumor regression grade locally. The change in OPG during NACT was unrelated to the diarrhea scores. However, at each serum sampling point during the active neoadjuvant therapy, a correlation was found between the OPG level and the actual monocyte count [125]. Since OPG is expressed by monocyte-derived dendritic cells [144], these observations suggest that the study treatment might have affected the dendritic cell pool.

#### 5.2.4. FLT3LG

This is a hematopoietic factor that can activate functionally mature dendritic cells [145,146,147] and is a potent growth factor for a rare subset of professional antigen-presenting cells that stimulate cytotoxic T-cells [148]. FLT3LG may reflect recovery from chemotherapy-induced myelosuppression [149,150] and in a preclinical model, it mediated radiation abscopal effects [59]. When administered to patients with metastatic CRC, it has led to expansion of the dendritic cell population both in the tumor periphery and systemically [59,151].

We showed that circulating FLT3LG increased following NACT and the sequential CRT and that the post-NACT increase was associated with favorable survival outcome. We further observed that patients who had an oxaliplatin dose intensity under CRT that maintained compliance to the radiotherapy protocol, had low probability of metastatic progression after the definitive surgery [100]. Our interpretation was that oxaliplatin, in the short-course NACT and then dosed at an adapted intensity under CRT, caused repetitive myelosuppression and elevated circulating FLT3LG as a rescue response, which may have resulted in an increase of functionally active immune cells, enabling eradication of occult microscopic tumor cells at distant sites (abscopal effect) in a patient population prone to metastatic progression. The data also supported the notion in which cytotoxic therapies, after transient immune suppression, may reset the immune system to reinstate immune surveillance by creating new immune cell repertoires [152,153]. The increase in circulating FLT3LG in response to NACT and CRT was also validated in an independent cohort of LARC patients [100].

### 5.3. New Insights and New Concepts

**Figure 2:**
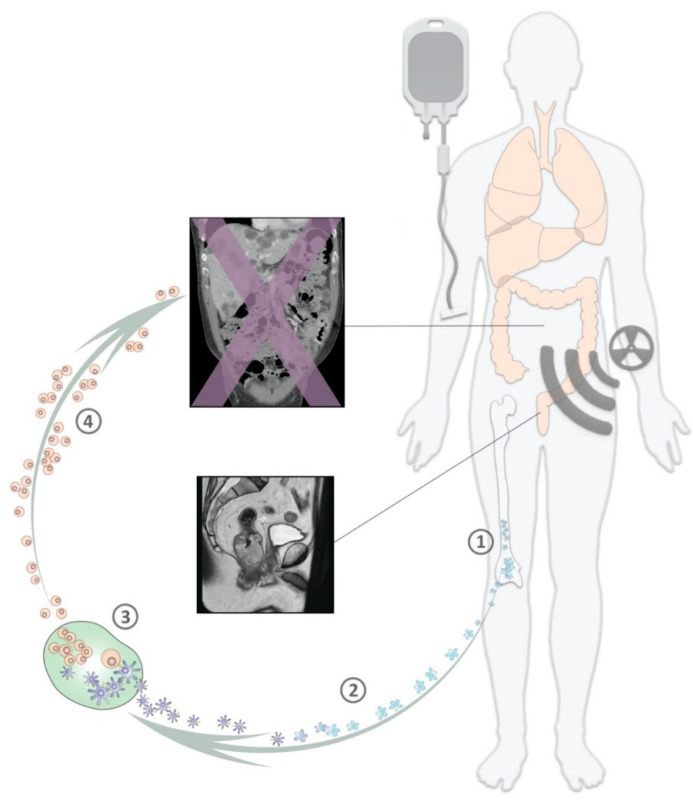
Tumor-defeating immunity under neoadjuvant cytotoxic therapy in high-risk rectal cancer. Short-course induction chemotherapy and sequential chemoradiotherapy in locally advanced rectal cancer cause repetitive myelosuppression and a resulting replenishment of the hematopoietic cell pool (1) when both treatment modalities contain oxaliplatin. The enhanced recruitment of maturing dendritic cells (2) enables the presentation of tumor antigens, released from the dying tumor, to cytotoxic T-cells (3), which after clonal expansion (4) may eliminate microscopic tumor cells at distant sites in the patient at high risk of metastatic progression.

In this LARC study, the initial modality of the oxaliplatin-containing neoadjuvant therapy course, consisting of two well-tolerated cycles of the Nordic FLOX regimen [120], led to a considerable reduction of the tumor burden [123], which comprised eradication of hypoxic tumor components [124] and invasive attributes of the TME [119]. Moreover, the induction NACT invoked systemic antitumor immunity that was maintained by the sequential oxaliplatin-containing CRT [100,125] (Figure 2). These observations suggest that CRC can be transformed into an immunogenic condition by oxaliplatin, and thereby large patient populations may benefit from the addition of ICB therapy in order to improve the outcome of oxaliplatin-based standard-of-care regimens.

## 6. Opportunities—Ongoing Combined-Modality Studies

Conventional chemotherapy and radiotherapy are particularly appealing now that more is known about their effects on the immune system, and about the fact that dose and timing of the administration play a decisive role. From this, a next step in CRC management will be the rational combination of cytotoxic agents with ICB therapy in patients with locally advanced or advanced pMMR CRC (Table 1), as for example illustrated by the NSABP FR-2 (NCT03102047) and METIMMOX (NCT03388190) trials. In the former, a PD-L1 inhibitor is administered following CRT completion in patients with pMMR LARC. If CRT invokes ICD, the sequential ICB therapy may improve the primary outcome measure, the neoadjuvant rectal score, which is a composite short-term surrogate endpoint for long-term survival in rectal cancer [154].

**Figure 3:**
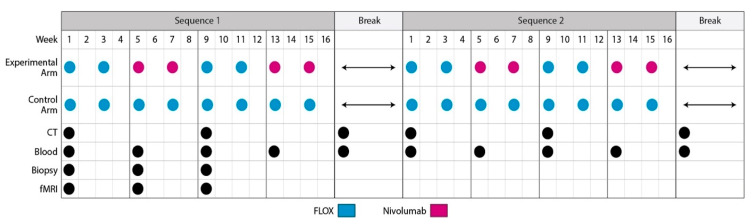
The METIMMOX study flow chart. Each treatment arm consists of intermittent sequences of active therapy over 8 cycles (16 weeks), before break until disease progression, when the therapy is reintroduced and administered for another 8 cycles before a new break. This “go-and-stop” schedule is continued until disease progression on ongoing therapy (defining progression-free survival in terms of failure of treatment strategy), intolerable toxicity, withdrawal of consent, or death, whichever occurs first. Circles indicate study visits with appendant activities. During a break period, radiographic assessment (CT), blood biobanking, and visits are done every 8 weeks until start of a new therapy sequence. Adverse events are recorded at each visit. Quality-of-life assessments are done before Week 1, at the end of Sequence 1 (Week 16), and at disease progression on study treatment. Timing for blood and biopsy biobanking and functional magnetic resonance imaging (fMRI) recordings is indicated.

In our own study, METIMMOX: Colorectal Cancer Metastasis—Shaping Antitumor Immunity by Oxaliplatin, patients with unresectable and previously untreated metastatic pMMR CRC are randomized to either the standard-of-care Nordic FLOX regimen or repeat sequential two FLOX cycles and two cycles of the PD-1 inhibitor nivolumab (Figure 3). The primary objective is to compare PFS in the experimental versus standard-of-care arm. Secondary objectives are to compare safety, tolerability, quality-of-life, and OS in the two study arms, and the tertiary objective is to compare costs for the resource use. Exploratory objectives are to develop circulating, tissue-based, and functional magnetic resonance imaging indicators of TME immune responses within a companion biomarker program.

## 7. Conclusions

As discussed in this review, there is clear evidence that the effects of cytotoxic therapies extend beyond the shrinking of the tumor masses and involve factors with critical roles in tumor-targeting immunity. Investigations of these factors, as they appear systemically and intratumorally over the course of treatment, will lead to a better understanding of the immune-modulating effects of conventional cancer therapies and match the accumulating knowledge on the immunologic characteristics of individual tumors. In CRC, with a complex TME consisting of hypoxic regions with highly invasive propensity and immune tolerance that pose therapeutic challenges, the next therapeutic milestone is likely found here.

## Figures and Tables

**Table 1:** Trials (recruiting as per July 2020) that investigate combined-modality cytotoxic agents and immune checkpoint inhibitors in colorectal cancer proficient in the DNA mismatch-repair function.

Condition	Study Agents	Identifier	Main Study Endpoints	Phase
Locally advanced rectal cancer	Radiation Fluoropyrimidine Durvalumab	NCT03102047	Efficacy and tolerability of immune checkpoint blockade following completion of neoadjuvant chemoradiotherapy	2
Locally advanced rectal cancer	Radiation Fluoropyrimidine Atezolizumab	NCT03127007	Efficacy and tolerability of concomitant immune checkpoint blockade under neoadjuvant chemoradiotherapy	1/2
Oligometastatic colorectal cancer	Radiation Toripalimab	NCT03927898	Efficacy and tolerability of immune checkpoint blockade following first-line chemotherapy and stereotactic body radiotherapy	2
Metastatic colorectal cancer	Radiation Nivolumab Ipilimumab	NCT03104439	Efficacy of dual immune checkpoint blockade under palliative radiotherapy	2
Metastatic colorectal cancer	Oxaliplatin Fluoropyrimidine Nivolumab	NCT03388190	Efficacy and tolerability of repeat sequential chemotherapy and immune checkpoint blockade	2
Metastatic colorectal cancer	Oxaliplatin Irinotecan Fluoropyrimidine Binimetinib Pembrolizumab	NCT03374254	Efficacy and tolerability of concomitant chemotherapy, inhibitor of mitogen-activated protein kinase 1 and 2, and immune checkpoint blockade	1
Metastatic colorectal cancer	Oxaliplatin Irinotecan Fluoropyrimidine Bevacizumab Atezolizumab	NCT03721653	Efficacy and tolerability of concomitant combination chemotherapy, angiogenesis inhibitor, and immune checkpoint blockade	2
Metastatic colorectal cancer	Oxaliplatin Fluoropyrimidine Durvalumab Tremelimumab	NCT03202758	Safety, efficacy, and tolerability of concomitant chemotherapy and dual immune checkpoint blockade	1/2
Locally advanced or metastatic colorectal cancer	Fluoropyrimidine Bevacizumab Pembrolizumab	NCT03396926	Efficacy of concomitant chemotherapy, angiogenesis inhibitor, and immune checkpoint blockade	2

## Abbreviations

CA9	carbonic anhydrase 9
CLM	colorectal liver metastasis
CMS	consensus molecular subtype
CRC	colorectal cancer
CRT	chemoradiotherapy
dMMR	deficient MMR function
FLT3LG	fms-related tyrosine kinase 3 ligand
HAI	hepatic artery infusion
HMGB1	high-mobility group box-1
ICB	immune checkpoint blockade
ICD	immunogenic cell death
LARC	locally advanced rectal cancer
MDSC	myeloid-derived suppressor cells
MMP9	matrix metallopeptidase 9
MMR	DNA mismatch-repair
MSI	microsatellite-instable
NACT	neoadjuvant chemotherapy
OPG	osteoprotegerin
OS	overall survival
PD-1	programmed death receptor 1
PD-L1	PD-1 ligand
pMMR	proficient MMR function
PFS	progression-free survival
TME	tumor microenvironment
